# Ⅰb期肺癌术后辅助化疗高风险因素分析

**DOI:** 10.3779/j.issn.1009-3419.2014.05.09

**Published:** 2014-05-20

**Authors:** 锋 毛, 雁 潘, 子明 李, 明辉 蔡, 屠阳 申

**Affiliations:** 200030 上海，上海交通大学附属胸科医院/上海市肺部肿瘤临床医学中心胸外科 Department of Thoracic Surgery, Shanghai Chest Hospital, Shanghai Jiao Tong University, Shanghai 200030, China

**Keywords:** 肺肿瘤, 手术, 脉管癌栓, 预后, Lung neoplasms, Operation, Vessel carcinoma embolus, Prognostic factor

## Abstract

**背景与目的:**

Ⅰb期肺癌患者术后2年内转移和复发率超过35%，预后仍较差，而该期患者术后是否需要辅助化疗依然存在争议。本探索研究影响早期非小细胞肺癌手术预后的相关临床病理学因素，探讨术后辅助化疗的高风险指征。

**方法:**

281例接受完全性切除的Ⅰb期非小细胞肺癌患者，依据*Cox*回归模型进行预后多因素分析，采用*Kaplan-Meier*方法进行生存分析。

**结果:**

单因素分析显示：①存在淋巴管或血管内癌栓、低分化肿瘤、肿瘤位于中下叶者预后较差（*P* < 0.05）；②患者的年龄、性别、病理类型、肿瘤侵犯胸膜、术后辅助化疗与术后生存无明显关系（*P* > 0.05）。多因素分析显示：血管内癌栓和肿瘤低分化是影响患者生存率的主要因素。

**结论:**

Ⅰb期非小细胞肺癌患者肿瘤细胞分化程度及脉管内癌栓是影响手术预后及生存率的重要因素，低分化肿瘤和脉管癌栓可作为术后辅助化疗的指征之一。

## Abstract (en)

**Background and objective:**

More than 35% Ib stage lung cancers have metastasised or recurrenced after operation, whose prognosis still remain poor. There're much controversy over the necessity of adjuvant chemotherapy to them. This aim of this study is investigated the clinical and pathological characters influencing prognosis of the stage Ib non-small cell lung cancer (NSCLC) and to explore the indication of postoperative chemotherapy.

**Methods:**

NSCLC patients (281 cases) who underwent lobectomy were examined. *Cox* proportional-hazards ratios were used to identify independent prognostic factors for survival. *Kaplan-Meier* survival curves were calculated to estimate survival rates.

**Results:**

*Kaplan-Meier* analysis show that, cancerous embolus in the blood vessel or lymphatic vessel, histologic grade and tumor location were remarkerbly associated with mortality risk (*P* < 0.05). The poor histologic grade and cancerous embolus in the blood vessel were closely associated with increased mortality risk on multivariate analysis in stage Ib NSCLC.

**Conclusion:**

The low histologic grade and cancerous embolus in the blood vessel or lymphatic vessel are closely correlated with survival in the stage Ib NSCLC and can be an index for the postoperative chemotherapy.

全球范围内，肺癌总体发病率及病死率位居各类癌症之首^[1]^，非小细胞肺癌（non-small cell lung cancer, NSCLC）占肺癌的80%-85%^[2]^，手术切除是早期肺癌的首选治疗方法。Ⅰb期肺癌患者术后5年生存率为60%左右，但术后2年内转移和复发率也达到38%，预后仍然较差^[3, 4]^，而该期患者术后是否需要辅助化疗仍存在争议^[5, 6]^。本研究收集我院早期肺癌手术患者，依据2009年第七版UICC TNM标准重新分期，选择其中Ⅰb期者研究相关手术预后的临床病理因素，并探讨影响该期患者长期生存的负面因素，为早期肺癌术后辅助化疗的指征提供理论依据。

## 1 资料与方法

### 1.1 患者资料

2002年1月-2004年12月，我院共进行肺癌手术1, 518例。术前依据患者胸部增强CT、颅脑CT、全身骨扫描和腹部B超等判断为Ⅰa-Ⅲa期。术后病理证实Ⅰ期肺癌患者398例。

### 1.2 研究对象

在398例Ⅰ期肺癌患者中，筛选符合条件患者入组研究。入组标准：①术前未行辅助放化疗者；②完全性肺癌切除术者；③按UICC2009（第七版）TNM分期中T分期标准判断，术后属于T2aN0M0者；④肿瘤细胞分化程度按低、中低、中、中高和高分化5等级区分；⑤有完整5年随访资料者。按上述标准，共计入组Ⅰb期肺癌患者281例，其中男158例，女123例，平均年龄59.15岁。

### 1.3 研究方法

281例Ⅰb期患者随访的术后生存时间按月计算，死亡患者以手术日距死亡日的差值计算，存活患者以手术日距末次随访日的差值计算。随访数据源于上海疾病控制中心，所有患者随访至2009年12月31日。采用SPSS 19.0软件进行统计分析，用*Kaplan-Meier*法进行生存分析，用*Cox*模型进行多因素生存分析，*Log-rank*检验进行组间生存率的比较，检验水准α= 0.05，以*P* < 0.05为差异有统计学意义。

## 2 结果

### 2.1 临床结局

手术部位：左上叶82例，左下叶40例，右上叶83，右中叶15例，右下叶50例，右中下叶11例；术中输血65例，未输血216例；病理类型：腺癌184例，鳞癌69例，鳞腺混合型28例；脏层胸膜受侵240例，无脏层胸膜受侵41例；肿瘤细胞分化程度：低分化21例，中低分化22例，中分化63例，中高分化54例，高分化53例，分化未明68例；肿瘤最大直径≤2 cm 54例，2 cm < 直径≤3 cm 101例，3 cm < 直径≤5 cm 126例；存在淋巴管癌栓12例，血管内癌栓15例。

### 2.2 生存分析

281例患者术后5年累积生存率71%，中位生存时间87.857。男女患者5年生存状况见图 1A。

**1:**
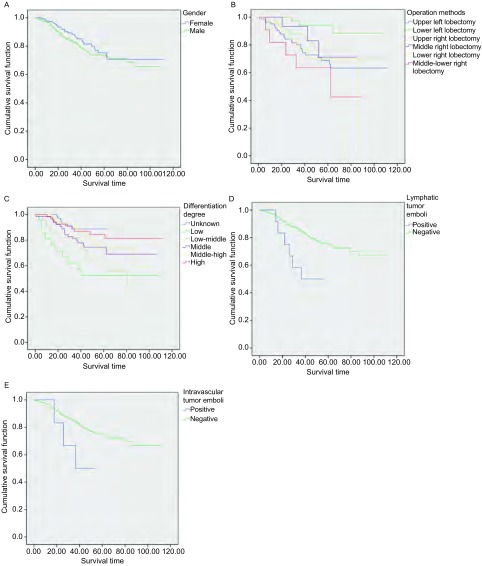
生存分析。A：281例Ⅰb期肺癌患者生存时间分析；B：手术方式对患者生存的影响；C：肿瘤细胞分化程度对患者生存的影响；D：淋巴管内癌栓对患者生存的影响；E：血管内癌栓对患者生存的影响。 Survival time analysis. A: survival time analysis of 281 patients with stage Ⅰb lung cancer; B: the influence of operation methods for patients survival; C: the impact of tumor cell differentiation degree on patients survival; D: the impact of lymphatic tumor emboli on patients survival; E: the impact of intravascular tumor emboli on patients survival.

### 2.3 单因素分析

用*Kaplan-Meier*法进行生存分析结果见表 1。

**1:** 281例Ⅰb期肺癌患者生存单因素分析结果 Results of univariate analysis of postoperative survival in the 281 patients with stage Ⅰb lung cancer

Clinical features		Counts	Percentage (%)	The median survival	Standard error (E)	Log-rank/*χ*^2^	*P*
Gender						0.389	0.533
	Male	158	56.23	84.744	3.191		
	Female	123	43.77	90.152	3.745		
Blood transfusion						0.046	0.830
	Positive	65	23.13	86.732	4.776		
	Negative	216	76.87	85.616	2.718		
Operation methods						12.74	0.026^*^
	Upper left lobe	82	29.18	82.597	4.842		
	Lower left lobe	40	14.23	100.111	3.752		
	Upper right lobe	83	29.54	88.956	4.163		
	Middle right lobe	15	5.34	72.129	6.269		
	Lower right lobe	50	17.79	86.270	4.334		
	Middle-lower right lobe	11	3.91	62.430	28.016		
Pathological classification						0.399	0.819
	Adeno	184	65.48	87.153	3.353		
	Squamous	69	24.56	86.796	4.693		
	Adeno-squamous	28	9.96	72.088	6.732		
Completed resection						0.086	0.769
	Negative	74	26.33	86.855	4.682		
	Positive	207	73.67	87.585	2.896		
Pleural invasion						0.603	0.437
	Negative	41	14.59	75.441	5.521		
	Positive	240	85.41	88.306	2.793		
Tumor diameter						5.006	0.082
	d≤2 cm	54	19.22	83.671	5.032		
	2 cm < d≤3 cm	101	35.94	90.290	3.418		
	3 cm < d≤5 cm	126	44.84	81.492	3.999		
Differentiation degree						12.554	0.028^*^
	Unknown	68	24.20	59.795	1.634		
	Low	21	7.47	66.008	10.037		
	Low-middle	22	7.83	80.430	19.739		
	Middle	63	22.42	83.516	4.659		
	Middle-high	54	19.22	82.105	4.350		
	High	53	18.86	97.002	4.551		
Lymphatic tumor emboli						5.950	0.015^*^
	Positive	12	4.27	36.530	4.866		
	Negative	269	95.73	88.857	2.579		
Intravascular tumor emboli						10.273	0.001^**^
	Positive	15	5.34	38.544	4.385		
	Negative	266	94.66	89.293	2.579		
^*^P < 0.05; ^**^P < 0.01.							

#### 2.3.1 手术方式对生存率的影响

本组病例中，左上叶切除术者82例，左下叶切除术者40例，右上叶切除术者83，右中叶切除术者15例，右下叶切除术者50例，右中下叶切除术者11例；其中右中下叶切除术者术后中位生存期为62.43月，较其它术式者预后有明显差异（*P* < 0.05），见图 1B。

#### 2.3.2 肿瘤细胞分化程度对生存率的影响

本组病例中，低分化21例，中低分化22例，中分化63例，中高分化54例，高分化53例，分化未明68例，其中低分化者较其他分化类型者预后差（*P* < 0.05），见图 1C。

#### 2.3.3 淋巴管癌栓对生存率的影响

本组病例中，存在淋巴管癌栓者12例，预后较无癌栓者差（*P* < 0.05），见图 1D。

#### 2.3.4 血管癌栓对生存率的影响

本组病例中，存在脉管癌栓者15例，预后较无癌栓者差（*P* < 0.05），见图 1E。

### 2.4 *Cox*模型多因素生存分析

将患者年龄、性别、手术方式、术中是否输血、肿瘤最大径、是否侵犯胸膜、肿瘤细胞分化程度、是否合并淋巴管、血管、脉管癌栓等代入*Cox*模型进行多因素分析，结果显示手术方式、肿瘤细胞分化程度、脉管内癌栓是是影响患者术后生存的独立预后因素。见表 2。

**2:** Cox模型多因素生存分析 Cox survival analysis model

Clinical features		B	SE	*P*	Risk rito Exp(B)	95%CI to Exp(B)
Gender		0.115	0.313	0.713	1.122	0.608-2.07
Blood transfusion		-0.668	0.342	0.051	0.513	0.262-1.002
Operation methods				0.007^**^		
	Upper left	-0.082	0.557	0.884	0.922	0.309-2.746
	Lower left	-2.198	0.774	0.005^**^	0.111	0.024-0.507
	Upper right	-0.958	0.569	0.092	0.384	0.126-1.170
	Middle right	-1.019	0.791	0.198	0.361	0.077-1.700
	Lower right	-0.304	0.584	0.602	0.738	0.235-2.316
Pathological classification				0.753		
Adeno	Type (1)	0.116	0.443	0.793	1.123	0.471-2.676
Squamous	Type (2)	-0.149	0.450	0.74	0.861	0.356-2.082
Completed resection		-0.285	0.338	0.399	0.752	0.388-1.458
	Pleural invasion	-0.068	0.388	0.861	0.934	0.436-2.001
Tumor diameter				0.075		
	d≤2 cm	-0.091	0.248	0.715	0.913	0.561-1.486
	2 cm < d≤3 cm	-0.305	0.210	0.147	0.737	0.488-1.113
Differentiation degree				0.007^**^		
	Unknown	-0.686	0.594	0.248	0.504	0.157-1.613
	Low	1.117	0.373	0.003^**^	3.055	1.47-6.351
	Low-middle	0.857	0.353	0.015^*^	2.356	1.179-4.708
	Middle	-0.383	0.268	0.153	0.682	0.403-1.153
	Middle-high	-0.194	0.288	0.500	0.823	0.468-1.449
Lymphatic tumor emboli		-1.865	1.386	0.179	0.155	0.01-2.345
Vascular tumor emboli		-1.455	0.740	0.049^*^	0.233	0.055-0.994
^*^P < 0.05; ^**^P < 0.01.						

## 3 讨论

目前早期肺癌的治疗原则仍是早期手术治疗，但手术后仍有60%患者存活不满5年，患者预后受多种因素的影响，因此以手术为主的多学科治疗受到人们的重点关注。本研究综合分析了影响Ⅰb期肺癌患者长期生存预后的临床、病理及治疗等因素，并进行了预后因素的*Cox*回归分析，以便指导临床采取更科学的治疗手段以延长患者的生存时间。

IASLC（International Association for the Study of Lung Cancer）于2007年在世界肺癌大会上提出了对第7版肺癌TNM分期的修改建议对规范肺癌的诊治及临床研究起到了很重要的作用^[7]^。基于*Log-rank*分析确定的最佳切入点，IASLC对肺癌的分期结果显T1肿瘤应分为2个亚组。因此，T1期肺癌分为了2个不同预后组：≤2 cm（T1a）和 > 2 cm但≤3 cm（T1b）。在符合手术完全切除、病理分期为N0（R0 pN0）的患者中，按照第6版肺癌分期标准并根据肿瘤大小进行调整，p T1a（肿瘤最大直径≤2 cm，*n*=1, 816）、p T1b（3 cm≥肿瘤最大直径 > 2 cm，*n*=1, 653）、p T2a（5 cm≥肿瘤最大直径 > 3 cm，*n*=2, 822）、p T2b（7 cm≥肿瘤最大直径 > 5 cm，*n*=825）、p T2c（肿瘤最大直径 > 7 cm，*n*=364）。本研究中按照新的T分期进行分析，分析结果表明患者的5年生存率71%，与文献相似，中位生存期87个月，高于旧分期的数据，这可能是因为剔除了部分被新分期划分到Ⅱa期的患者，因此有充分确实的信息证明第7版肺癌TNM分期的建议值得采纳。

在肿瘤完全切除的NSCLC患者中，辅助化疗已被证实能够改善早期肺癌患者的生存^[8, 9]^。但是目前关于Ⅰb期患者仍然有很大的争议。2004年在美国临床肿瘤年会上报道美国CALGB 9633实验组观察了344例Ⅰb期患者完全切除术后辅助卡铂加紫杉醇化疗，不加辅助放疗，最长随访4年，平均随访34个月，生存率增加12%（*P* < 0.028）^[10]^。但到了2006年美国临床肿瘤年会，该研究^[11]^第5年的随访资料显示研究组与对照组的生存曲线又逐渐归并一致（5年生存率60% *vs* 57%，*P*=0.32），这使得Ⅰ期NSCLC术后辅助化疗的作用再次成为不确定因素，进一步的分层分析发现术后辅助化疗仅对肿瘤≥4 cm的Ⅰb期病变有临床意义，因此需要研究者对于哪些是高危患者进一步甄别。2007年，美国和加拿大两大肿瘤研究中心的研究报告提出了完全切除的NSCLC的术后治疗指导方针^[12]^，对于Ⅰb期患者尽管一些试验证实术后化疗可能有一定的益处，但不建议常规使用。2011版NCCN提出NSCLC术后的高危因素包括低分化、神经内分泌瘤、侵犯脉管、楔形切除、肿瘤 > 4 cm和脏层胸膜受累等。T2abN0且切缘阴性的患者一般仅接受观察，有以上高危特征的患者推荐行辅助化疗；T2abN0患者如切缘阳性应该接受再切除术加化疗或化放疗加化疗。对于手术切缘阴性的Ⅱ期病变，推荐化疗（1类）加或不加放疗（放疗为3类）。如果Ⅱ期患者切缘阳性，可选治疗方法包括再次手术切除加化疗或化放疗联合化疗^[13]^。

本研究中无癌栓患者的死亡风险与有癌栓者相比：淋巴管0.155、血管0.233，血管癌栓对患者预后有统计学差异，这一结果提示对于这部分患者可能需要进行辅助化疗。考虑到血管内的癌栓可能是癌细胞的扩散的信号，与微转移存在一定的联系，术后细致甄别病理切片中血管癌栓就显得极为重要，其与骨骼微转移的关系有待进一步的研究，需要更大样本和更长随访时间的研究支持该结果。尽管如此，血管癌栓作为影响预后的临床病理指标已受到重视，不同地域多个中心的研究都把血管受癌栓累及作为完全性手术切除的早期肺癌患者需要辅助化疗的指证^[14, 15]^。更有研究^[16]^认为，血管受癌栓累及可作为TNM分期系统中T2期的独立分期因素。

同时本研究发现Ⅰb期非小细胞肺癌患者手术方式与细胞分化程度是影响预后及生存的重要因素，其中肿瘤低分化以及两叶切除预后较单叶切除差。Ou^[17]^研究了1989年-2003年间19, 702例Ⅰ期肺癌患者，在单因素分析中发现中下叶肿瘤比上叶预后差（*P*=0.048），本研究结果与Ou的结论相似，可能是由于上叶肺癌不容易转移到纵隔淋巴结是导致患者预后较好的原因。另外显而易见的是NSCLC的分化程度与预后有很大关系，分化越低恶性程度越高，患者的生存率越低，在本试验中同样得到证实。对具备以上高危因素的患者可以考虑为术后辅助化疗的对象。

虽然本文为回顾性研究，但是为治疗策略的制订和进一步前瞻性研究的设计提供了重要的参考依据。Ⅰb期完全切除的患者如存在NCCN定义的高危因素，术后辅助化疗应能使患者有更大的生存获益。

## References

[1] Jemal A, Bray F, Center MM (2011). Global cancer statistics. CA Cancer J Clin.

[2] Beasley MB, Brambilla E, Travis WD (2005). The 2004 World Health Organization classification of lung tumors. Semin Roentgenol.

[3] Kelsey CR, Marks LB, Hollis D (2009). Local recurrende after surgery for early stage lung cancer: an 11-year experience with 975 patients. Cancer.

[4] Su S, Scott WJ, Allen MS (2014). Patterns of survival and recurrence after surgical treatment of early stagenon-small cell lung carcinoma in the ACOSOG Z0030 (ALLIANCE) trial. Thorac Cardiovasc Surg.

[5] Arriagada R, Dunant A, Pignon JP (2010). Long-term results of the international adjuvant lung cancer trial evaluating adjuvant cisplatin-based chemotherapy in resected lung cancer. J Clin Oncol.

[6] Ioannidis G, Georgoulias V, Souglakos J (2011). How close are we to customizing chemotherapy in early non-small cell lung cancer?. Ther Adv Med Oncol.

[7] Rami-Porta R, Ball D, Crowley J (2007). The IASLC Lung Cancer Staging Project: proposals for the revision of the T descriptors in the forthcoming (seventh) edition of the TNM classification for lung cancer. J Thorac Oncol.

[8] Besse B, Le Chevalier T (2012). Developments in the treatment of early NSCLC: when to use chemotherapy. Ann Oncol.

[b9] 9Williams CD, Gajra A, Ganti AK, *et al*. Use and impact of adjuvant chemotherapy in patients with resected non-small cell lung cancer. Cancer, 2014. [Epub ahead of print].

[10] Pisters KM, Evans WK, Azzoli CG (2007). Cancer Care Ontario and American Society of Clinical Oncology adjuvant chemotherapy and adjuvant radiation therapy for stages Ⅰ-ⅢA respectable non small-cell lung cancer guideline. J Clin Oncol.

[11] Pignon JP, Tribodet H, Scagliotti GV (2008). Lung adjuvant cisplatin evaluation: a pooled analysis by the LACE Collaborative Group. J Clin Oncol.

[12] Strauss GM, Herndon JE, Maddaus MA (2006). Adjuvant chemotherapy in stage IB non-small cell lung cancer (NSCLC): Update of Cancer and Leukemia Group B (CALGB) protocol 9633. ASCO Annual Meeting Proceedings, Atlanta, Georgia, USA. J Clin Nocol.

[13] Ettinger DS, Akerley W, NCCN (National Comprehensive Cancer Network) (2013). Non-small cell lung cancer, version 2.2013. J Natl Compr Canc Netw.

[14] Tao H, Hayashi T, Sano F (2013). Prognostic impact of lymphovascular invasion compared with that of visceral pleural invasion in patients with pN0 non-small-cell lung cancer and a tumor diameter of 2 cm or smaller. J Surg Res.

[15] Salvati F (2013). Lymphovascular invasion in non-small-cell lung cancer. J Thorac Oncol.

[16] Higgins KA1, Chino JP, Ready N (2012). Lymphovascular invasion in non-small-cell lung cancer: implications for staging and adjuvant therapy. J Thorac Oncol.

[17] Ou SH, Zell JA, Ziogas A (2007). Prognostic factors for survival of stage Ⅰ nonsmall cell lung cancer patients: a population-based analysis of 19, 702 stage Ⅰ patients in the California Cancer Registry from 1989 to 2003. Cancer.

